# Impairment of Lysosomal Activity as a Therapeutic Modality Targeting Cancer Stem Cells of Embryonal Rhabdomyosarcoma Cell Line RD

**DOI:** 10.1371/journal.pone.0110340

**Published:** 2014-10-20

**Authors:** Manuela Salerno, Sofia Avnet, Gloria Bonuccelli, Shigekuni Hosogi, Donatella Granchi, Nicola Baldini

**Affiliations:** 1 Orthopaedic Pathophysiology and Regenerative Medicine Unit, Istituto Ortopedico Rizzoli, Bologna, Italy; 2 Department of Biomedical and Neuromotor Sciences, University of Bologna, Bologna, Italy; 3 Department of Molecular Cell Physiology, Graduate School of Medical Science, Kyoto Prefectural University of Medicine, Kyoto, Japan; University of L’Aquila, Italy

## Abstract

Rhabdomyosarcoma is the most frequent soft tissue sarcoma in children and adolescents, with a high rate of relapse that dramatically affects the clinical outcome. Multiagent chemotherapy, in combination with surgery and/or radiation therapy, is the treatment of choice. However, the relapse rate is disappointingly high and identification of new therapeutic tools is urgently needed. Under this respect, the selective block of key features of cancer stem cells (CSC) appears particularly promising. In this study, we isolated rhabdomyosarcoma CSC with stem-like features (high expression of NANOG and OCT3/4, self-renewal ability, multipotency). Rhabdomyosarcoma CSC showed higher invasive ability and a reduced cytotoxicity to doxorubicin in comparison to native cells, through a mechanism unrelated to the classical multidrug resistance process. This was dependent on a high level of lysosome acidity mediated by a high expression of vacuolar ATPase (V-ATPase). Since it was not associated with other paediatric cancers, like Ewing’s sarcoma and neuroblastoma, V-ATPase higher expression in CSC was rhabdomyosarcoma specific. Inhibition of lysosomal acidification by the V-ATPase inhibitor omeprazole, or by specific siRNA silencing, significantly enhanced doxorubicin cytoxicity. Unexpectedly, lysosomal targeting also blocked cell growth and reduced the invasive potential of rhabdomyosarcoma CSC, even at very low doses of omeprazole (10 and 50 µM, respectively). Based on these observations, we propose lysosome acidity as a valuable target to enhance chemosensitivity of rhabdomyosarcoma CSC, and suggest the use of anti-V-ATPase agents in combination with standard regimens as a promising tool for the eradication of minimal residual disease or the prevention of metastatic disease.

## Introduction

Rhabdomyosarcoma (RMS) is the most frequent solid tumor in childhood, histologically featuring different patterns of striated muscle differentiation and characterized by a very aggressive clinical behaviour [1]. Although the outcome of RMS patients has significantly improved over the past two decades based on the use of surgery and/or radiation therapy in combination with chemotherapy, relapses still occur in 30–40% of nonmetastatic patients. Moreover, about 15% of children with RMS show evidence of systemic disease at the time of diagnosis. These “high risk” subjects have limited treatment options and a poor prognosis [2], hence the urgent need to identify novel therapies based on a thorough knowledge of RMS biology.

An increasing body of evidence suggests that the inadequacy of current anticancer treatments to eradicate minimal residual disease and prevent relapse partly depends on their inability to target the subset of quiescent or low-proliferating tumor cells, known as cancer stem cells (CSC) [3]. CSC were first identified in leukemias [4] and subsequently described in several solid tumors [5], [6], [7], including sarcomas [8], [9], [10], [11], [12]. It is generally accepted that CSC efficiently initiate tumors, display stem-like features, and are responsible for local and systemic relapse due to unresponsiveness to anticancer agents [3]. A relationship between CSC and minimal residual disease has been reported [13], strongly suggesting that targeting these cells would hold a substantial potential to improve the outcome of patients treated with conventional anticancer agents. Indeed, CSC-like chemoresistant elements have already been identified also in RMS [14], [15].

Microenvironmental conditions are able to significantly modulate the stemness phenotype under physiological conditions as well as in cancer. Especially in the CSC niche, tumor cells respond to hypoxia by converting from aerobic respiration to glycolysis, which in turn produces lactic acid and causes local acidosis. The presence of such peculiar microenvironmental features has been related to the induction and maintenance of multipotency and stemness [16]. Extracellular acidosis is therefore a major player in the formation and maintenance of CSC, because, per se, is able to promote a stem-like phenotype. It is already known that malignant tumors, including sarcomas, are characterized by an acidic extracellular environment and that cancer cells usually contain a significant amount of acidic lysosomes. These features are in keeping with several features of malignancy, including invasiveness and resistance to anticancer therapies [17]. In fact, accumulation of basic drugs into acidic vesicles, or their neutralization through acidification of the extracellular environment is an effective mechanism of chemoresistance and may facilitate tumor invasion [18], [19]. For this reason, the CSC behaviour is influenced by biochemical and biophysical variables of the extracellular compartment.

In this study, we explored the role of lysosome acidification, sustained by the vacuolar (H+)-ATPase (V-ATPase) proton pump as a peculiar mechanism conferring a selective advantage to RMS CSC. We showed that V-ATPase is involved in invasiveness as well as in chemosensitivity of these cells, and that such features of malignancy may be completely reversed by blockage of the acidification process by therapeutic doses of proton pump inhibitors (PPI), suggesting the potential advantage of this class of drugs in combination with conventional anticancer agents to effectively target RMS CSC.

## Materials and Methods

### Cell lines

RD (RMS), MG-63 (osteosarcoma), SK-ES-1, A-673 (Ewing’s sarcoma, ES), SH-SY5Y, NB-100, and CHP-212 (neuroblastoma, NB) cell lines were purchased from the American Type Culture Collection (ATCC) and cultured in IMDM (Life Technologies), plus 20 U/mL penicillin, 100 mg/mL streptomycin, and 10% heat-inactivated fetal bovine serum (FBS) (complete medium). Multidrug resistant MG-63 cells were isolated by stepwise exposure to increasing doses of doxorubicin (DXR). The resultant MG-63 cell line that grew exponentially in the presence of 100 ng/mL of DXR was designated as the multidrug resistant variant MG-63-DXR100. MG-63-DXR100 were continuously exposed to 100 ng/mL of DXR to maintain the multidrug resistant phenotype [20].

### Sphere cultures

Sphere-forming cells were obtained as previously described [12]. Briefly, all cells were cultured in anchorage-independent conditions in DMEM:F12 medium with progesterone (20 nM), putresceine (10 mg/mL), sodium selenite (30 nM), apo-transferrin (100 µg/mL), and insulin (25 µg/mL) (Sigma-Aldrich) in low-attachment flasks (Nunc). Fresh human epidermal growth factor (20 ng/mL) and basic fibroblast growth factor (10 ng/mL) (PeproTech) were added twice a week until cells started to grow forming floating aggregates, named rhabdospheres. Cultures were expanded by mechanical dissociation of the spheres, followed by re-plating of cells and residual cell aggregates in complete medium. Only cultures able to growth under spherogenic colonies and displaying stem cell-related features were considered. The spheres were analysed under serum starved medium on low attachment substrates (non adherent condition) or adherent condition in the presence of serum, depending on the assay. All the isolated spheres were characterised for the expression of stem cell-related markers (NANOG and OCT3/4) and, only for RMS CSC, for the sphere forming efficiency and multipotency.

### Characterization of stem cell properties of rhabdospheres

The sphere-forming efficiency during serial passages was investigated by plating single cells from rhabdospheres at a density of 2,000 cells/mL in a 48-well plate to obtain new spheres. The total number of tumor spheres was counted, and the spheres dissociated to obtain the second and third generation of spheres. To evaluate multipotency, rhabdospheres were maintained as adherent cultures for 3 days and then seeded in different conditions. Briefly, for osteogenic differentiation, 100,000 cells were seeded in 6-well plates and grown in α-MEM supplemented with 10% FBS, 10 mM β-glycerophosphate, 10^−8^ M dexamethasone, and 50 mg/mL L-ascorbic acid 2-phosphate (Sigma). After 14 days, cells were fixed with 3.7% paraformaldehyde, and mineralization was evaluated by staining with 1% Alizarin Red S (pH 4.2; Sigma-Aldrich). For adipogenic differentiation, 100,000 cells were seeded in a 6-well plate and grown in DMEM high glucose (Lonza) supplemented with 10% FBS, 0.5 µM dexamethasone, 0.5 mM 3-isobutyl-1-methylxanthine, and 50 µM indomethacine (Sigma-Aldrich). After 17 days, cells were fixed and lipids stained with 0.3% Oil-Red-O. For chondrogenic differentiation, 500,000 cells were centrifuged in a 15 mL polypropylene conical tube and incubated in DMEM high glucose supplemented with 10% FBS, 10 mg/mL TGFβ1 (PeproTech), 100 µM L-ascorbic acid 2-phosphate, 6.25 µg/mL insulin, 40 µg/mL L-Proline (Sigma-Aldrich). After 3 weeks, sections derived from chondrogenic pellets were stained with Alcian Blue (pH 2.5).

### Gene expression

Gene expression was assessed to determine stem cell-related features and further characterize the sphere cultures. Total RNA was isolated from RMS, ES, and NB floating spheres or native cells with the NucleoSpin RNA II (Macherey-Nagel), and reverse transcribed. The expression of mRNA for OCT3/4 (NM_002701.4), NANOG (NM_024865.2), MDR1 (AF016535.1), ATPase V_0_c (NM_001101.2), matrix metalloproteinase (MMP) 9 (NM_004994.2), and CXC chemokine receptor-4 (CXCR4) (NM_001008540.1) was evaluated using a Light Cycler instrument (Roche Diagnostics), amplifying 1 µg of cDNA, and the Universal Probe Library (Roche Applied Science). Probes and primers were selected using web-based assay design software (ProbeFinder https://www.roche-applied-science.com): OCT3/4-f 5′-CTTCGCAAGCCCTCATTTC-3′-; OCT3/4-r 5′-GAGAAGGCGAAATCCGAAG-3′; NANOG-f 5′-ATGCCTCACACGGAGACTGT-3′; NANOG-r 5′-AGGGCTGTCCTGAATAAGCA-3′; MDR1-f 5′-GCCATCAGTCCTGTTCTTGG-3′; MDR1-r 5′-GCTTTTGCATACGCTAAGAGTTC-3′; ATPase V_0_c-f 5′-TTCGTTTTTCGCCGTCAT-3′; ATPaseV_0_c-r 5′-CCACTGGGATGATGGACTTC-3′; MMP9-f 5′-GAACCAATCTCACCGACAGG-3′; MMP9-r 5′-GCCACCCGAGTGTAACCATA-3′. The results were expressed as ratio between gene of interest and Tata Binding Protein (TBP, NM_003194.4; TBP-f 5′-TTGGGTTTTCCAGCTAAGTTCT-3′; TBP-r 5′-CCAGGAAATAACTCTGGCTCA-3′) as reference gene according to the 2^−ΔΔCT^ method [21].

### Western Blotting

Western blotting was carried out to detect the stem cell-related markers OCT3/4 and NANOG as well as MDR1, ATPase V_0_a1 subunit, and TBP. Floating sphere or native cells were lysated with hot lysis buffer (1% SDS, Tris pH 7.4 20 mM, 5% β-mercaptoethanol) for the analysis of OCT3/4 and NANOG, or with RIPA buffer (Tris pH 7.6 50 mM, NaCl 150 mM, Triton-X 100 5%, sodium deoxycholate 0.25%, EGTA pH 8 1 mM, NaF 1 mM, Sigma) supplemented with protease inhibitors (Roche), for the other proteins. Equal amounts of protein lysates were subjected to reducing SDS-PAGE on a polyacrylamide gel, followed by to immunoblotting analysis. Blots were probed with a sheep anti-OCT3/4 (Abcam), a mouse anti-NANOG (Abcam), a mouse anti-MDR1 (D-11, Santa Cruz), a rabbit anti-ATPase V_0_a1 (Abcam), or a rabbit anti-TBP (Santa Cruz) as reference. Incubation with horseradish peroxidase-conjugated secondary antibodies followed. The reaction was revealed by a chemiluminescence substrate (Pierce ECL Plus Western Blotting Substrate, Thermo Scientific). Immunoblot assays were repeated three times. The signal from each band was quantified by a dedicated software for densitometric evaluation (VisionWorksLS Analysis Software, Biospectrum, UVP).

### Immunofluorescence

For the staining of V-ATPase, rhabdospheres were allowed to adhere for 24****h in complete medium and fixed, then incubated with an anti-V-ATPase V_0_a1 polyclonal antibody (Sigma-Aldrich), followed by a secondary anti-rabbit antibody Alexa green 488****nm (Life Technologies). To observe vesicular localization of V-ATPase, actin cytoskeleton was co-stained using 0.5** µ**g/mL Phalloidin–Tetramethylrhodamine B isothiocyanate (TRITC) fluorescent dye (Sigma). Nuclei were counterstained with Hoechst 33258 (Sigma), and cells were observed by confocal microscopy (Nikon TI-E).

### Migration assay

The migration ability of rhabdospheres was evaluated by the Boyden chamber technique in comparison to RD. Briefly, single cells derived from RD or rhabdospheres after trypsinization were suspended in serum free medium containing 0.1% bovine serum albumin (BSA) and seeded in the upper compartment of a Boyden chamber (8-µm pore, Euroclone). The lower chambers contained 10% FBS in IMDM medium as a chemo-attractant. Cells were incubated at 37°C and allowed to migrate for 8 h. Cells attached to the upper surface of the filter were mechanically removed by scrubbing with cotton swabs. Chambers were stained in 0.5% crystal violet diluted in 100% methanol for 30 min, rinsed in water and examined under bright-field microscopy. Values for migration were obtained by counting 5 fields per membrane (X20 objective) and represent the average of four independent experiments.

### Invasion assay

MMP activity was quantified as previously described [22]. Briefly, cells from rhabdospheres and RD were seeded in 6-well plates (300,000 cells/well) and allowed to adhere. After 48 h, cells were washed and incubated at 37°C with 400 µL of phosphate buffered saline (PBS) for 3 h. PBS was then collected, centrifuged and the supernatant was used for the assay. Equal amounts of the supernatant were added to 100 µL of gelatin quenching (DQ Gelatin, Life Technologies) in a 96-well plate. Adherent cells were detached and counted. After 24 h at 37°C, the fluorescence emission was measured by a microplate reader (Tecan). Results were reported as the percentage of fluorescence emission with respect to acellular supernatant and normalized with the total number of cells. The experiment was repeated three times.

### Flow cytometry

Rhabdosphere cells and RD were dissociated by trypsin, counted and stained as follows. For the quantification of CD133 expression, cell suspensions were incubated with monoclonal CD133/1 antibody (AC133, Miltenyi Biotec) for 10****min, followed by 20****min incubation with anti-goat antibody Alexa green 488****nm (Life Technologies) at 4°C. For the evaluation of CXCR4 content, cell suspensions were stained with monoclonal CD184 (CXCR4)-PE (Miltenyi Biotec) for 10****min at 4°C. After staining, cells were then resuspended in PBS and analysed by a Coulter EPICS XL Flow Cytometer (Coulter Corporation, Beckman Coulter). Experiments were repeated three times.

### Drug sensitivity assay

Rhabdosphere cells and RD were plated in 6-well plates (200,000/well) and allowed to adhere. After 24 h, cells were incubated with DXR (10, 50, and 100 ng/mL; Sigma) or cisplatin (5, 10, and 100 µM; Sigma), and after additional 72 h the number of viable cells was evaluated by Trypan blue dye exclusion assay. The percentage of growth inhibition was calculated in respect to untreated cells. The drug half maximal effective concentration (EC_50_) for each cell line was calculated by the linear regression method. The experiment was repeated twice.

### DXR uptake

Rhabdosphere cells and RD were seeded into 8-well glass chamberslides and allowed to adhere. The cells were then exposed to DXR (10 µg/mL) for 15 min, washed, and directly observed by confocal microscopy. The level of nuclear DXR was quantified in at least 100 cells over different fields using the NIS-Elements Microscope Imaging Software (Nikon).

### Lysosome acidity evaluation

The emission spectrum of the pH-sensitive acridine orange (AO) was used to measure pH variations in acidic organelles [23], [24]. Rhabdosphere cells and RD were seeded into 6-well plates and allowed to adhere for 24 h in complete medium. The cells were then exposed to AO (1 µg/mL) for 10 min, washed, and directly observed by spectral confocal microscopy. To characterize the profile of AO emission spectra, the red band contribution (R%) within the whole emission spectrum was calculated as follows: R% = 100*I*_655_/(*I*_655_/*I*_530_) where *I*_655_ and *I*_530_ are the green (520–540 nm) and the red (645–665 nm) integrated emission intensities, respectively. The average R% was calculated for all the acidic organelles in 10 cells. The experiment was repeated twice.

### Cytosolic pH measurement

Cytosolic pH (pHc) of rhabdosphere cells and RD was measured by using carboxy-seminaphthorhodafluor-1 (carboxy-SNARF-1) (Molecular Probes). Cells were seeded into chamber slides and allowed to adhere for 24 h, and then 10 µM of carboxy-SNARF-1 were added to the culture medium for 30 min. The chamber slides were placed on the stage of the confocal microscope and allowed to adapt for at least 20 min before starting pHc measurements. The excitation laser beam of 514 nm (Arlaser) was directed to the sample via S Plan Fluor EL WD 40X lens (Nikon). The resulting fluorescence emission was collected at 644 nm and 594 nm. Several regions of interest (ROI) with a diameter of 1 µm were then randomly selected excluding nuclear regions. The emission ratio was calibrated using solutions (110 mM KCl, 25 mM KHCO_3_, 11 mM glucose, 1 mM MgCl_2_, 1 mM CaCl_2_, 10 mM HEPES) with varying pH levels and containing 10 µM nigericin (K+/H+ ionophore). The fluorescence emission ratio (644 nm/594 nm) was calculated and used to estimate pHc from the calibration curve. The experiment was repeated three times.

### Growth assays after lysosome targeting

To impair lysosome function, rhabdosphere cells and RD were treated using two different strategies. For the first one, cells were seeded in 6-well plates (200,000/well), allowed to adhere for 24 h in complete medium, and then treated with AO (0.1, 0.5, and 1 µg/mL; Sigma) that selectively accumulates into acidic lysosomes and affect cancer cell growth [25]. After 72 h, the number of viable cells was evaluated by dye exclusion assay. The experiment was repeated two times. The other strategy was assessed using the PPI omeprazole (OME), a drug that is known to inhibit V-ATPase activity [26]. Cell viability after OME treatment was determined using two different assays. a) For the indirect assay, rhabdospheres and RD, or cells representative of ES or NB histotype, were seeded in 96-well plates (8,000 cells/well) and allowed to adhere. After 24 h, the medium was changed with unbuffered RPMI with 10% FBS and treated with 10, 25, 50, and 100 µM of OME (Sigma-Aldrich). After 24, only for rhabdomyosarcoma cells, and 72 h for RMS, ES and NB cells, the viability was evaluated by an acid phosphatase (AP) assay. Briefly, the cells were washed and incubated at 37°C with 100 µL of buffer containing 0.1 M sodium acetate (pH 5.0), 0.1% Triton X-100, and 5 mM p-nitrophenil phosphate. After 3 h, the reaction was stopped with the addition of 10 µL of 1 N NaOH, and colour development was assayed at 405 nm using a microplate reader (Tecan) [23]. b) For the direct assay, rhabdospheres and RD were seeded in 6-well plates and allowed to adhere. After 24 h, 50 µM OME was added to the culture medium. After additional 24 h, the number of viable cells was determined by dye exclusion assay. The experiment was repeated twice.

### Apoptosis analysis

Rhabdosphere cells were allowed to adhere after 24 h in complete medium and exposed to 50 µM of OME in unbuffered RPMI with 10% FBS. After 24 and 48 h, cells were labelled with Hoechst 33258 and the presence of apoptotic bodies was detected under fluorescence microscopy.

### Cell cycle analysis

Rhabdospheres were exposed to 100 µM of OME in non adherent conditions at pH 6.8 for 24 h. The DNA content and bromodeoxyuridine (BrdU) incorporation during the S-phase were determined by simultaneous analysis of propidium iodide (PI) for the total DNA content and of FITC-conjugated anti-BrdU fluorescence. Briefly, cells were incubated with 40 µM 5-BrdU (Sigma) for 60 min at 37°C, washed and then 5×10^6^ single cells were fixed with 75% ethanol for 20 min at 4°C. Partial DNA denaturation was performed by incubating cells in HCl, followed by neutralization with Na tetraborate. Samples were then incubated with a mouse monoclonal anti-BrdU FITC antibody (BD Biosciences), washed, stained with 2.5 µg/mL PI (Sigma-Aldrich), and analysed. Monoparametric and biparametric analyses were performed using the WinMDI 2.7 software. The experiment was repeated twice.

### Effects of DXR on cell viability after OME treatment

Rhabdosphere cells let to adhere for 24 h in complete medium. The cells were then pre-treated with 10, 25, 50, and 100 µM of OME in unbuffered RPMI with 10% FBS. After additional 24 h, the cells were exposed to 50 and 25 ng/mL of DXR in buffered medium. After additional 48 h, cell viability was assessed by the AP assay, as previously described. Data were reported as cell survival in respect to untreated cells (set = 100%). The experiment was performed in quadruplicate.

### DXR uptake after OME treatment

Rhabdosphere cells let to adhere for 24 h in complete medium. The cells were then pre-treated with 10 and 20 µM of OME in unbuffered RPMI with 0.1% FBS for 1 h, and then exposed to 5 µg/mL of DXR for 15 min. The level of nuclear DXR was quantified in at least 100 cells over different fields, as previously described, and compared to that of untreated cells. The experiment was repeated twice.

### siRNA transfection

Specific gene silencing effect was obtained by siRNA technology associated with pipette-type electroporation. Briefly, rhabdosphere cells were trypsinized and 100 µL of cell suspension containing 2,000,000 cells and 1,6 nmol of specific siRNA (ON-TARGETplus Human ATP6V0C siRNA Smart pool, Dharmacon, Thermo Scientific) or control siRNA (siRNA ctr, ON-TARGETplus Non-targeting Control Pool, Dharmacon), or water (no siRNA) were transferred into a 1-mm cuvette (Neon Transfection System, Life Technologies). Electroporated cells were seeded in 6-well plates (500,000 cells/well) for RNA isolation and in a 96-well plate for the growth assay (15,000 cells/well). After 24 h, the reduction of mRNA level for the V_0_c ATPase was verified by Real Time PCR, as previously described. For the growth assay, the cells were exposed to 25 ng/mL of DXR in buffered medium and incubated for additional 48 h. Cell viability was assessed by the AP indirect assay, as previously described. The results were reported as percentage of cell survival *vs* control cells (no siRNA). The experiment was repeated twice.

### Inhibition of rhabdosphere cell migration by OME

Rhabdospheres were dissociated and seeded in 6-well plates let to adhere for 24****h in complete medium. To evaluate the migration ability, the cells were then pre-treated with 50** µ**M of OME in unbuffered RPMI with 10% FBS. After 24****h, viable cells were counted and then an equal number of untreated or OME-treated cells were seeded into the upper compartment of the Boyden chamber, as previously described. The experiment was repeated twice.

### Inhibition of rhabdosphere cell invasive potential by OME

To evaluate the invasive potential, rhabdosphere cells were let to adhere for 24 h in complete medium and then pre-treated with 50 µM of OME in unbuffered RPMI with 0.1% FBS for 3 h. The release of MMPs in the supernatant was quantified by gelatin quenching assay, as previously described, and normalised with the total number of viable cells. To evaluate the effect on the CXCR4-expressing population, rhabdospheres were seeded in sphere-forming condition at pH 6.8 and pre-treated with 100 µM of OME. After 48 h, the cells were subjected to viable counting and the CXCR4-positive fraction in the population of living cells (selected by the forward and side scattering parameters) was evaluated by flow cytometry, as previously described. The experiments were repeated twice.

### Statistical analysis

Due to the small number of observations, data were considered as not normally distributed. Values were expressed as means ± SE. Statistical analysis was performed with the StatView 5.0.1 software (SAS Institute Inc., Cary, NC). The nonparametric Mann-Whitney U test was used and p<0.05 was considered significant.

## Results

### Rhabdosphere formation and enrichment

**Figure 1:**
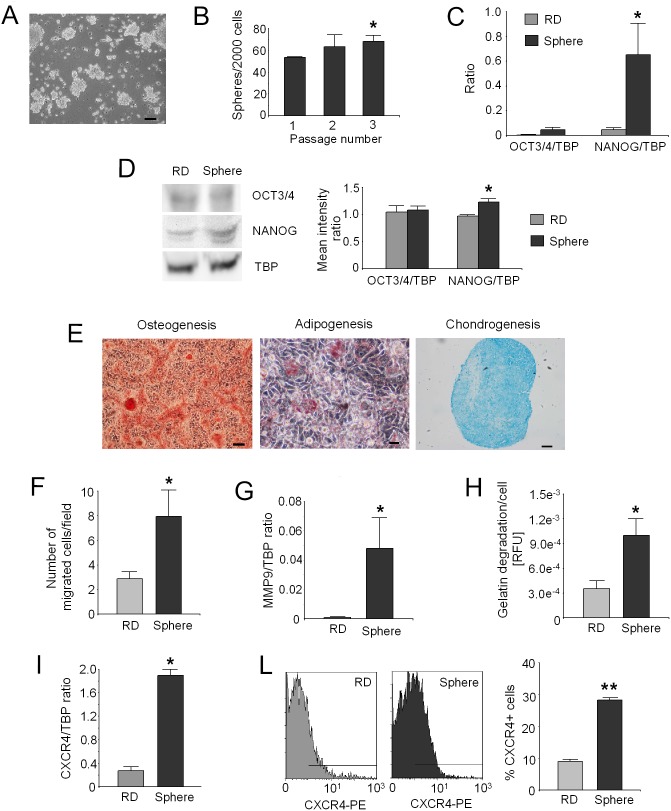
Stem cell-related properties, migration, and invasion ability of rhabdospheres. (A) Phase contrast pictures of rhabdospheres derived from RD grown in anchorage-independent condition, in serum-starved medium supplemented with bFGF and EGF. Representative image, scale bar 100 **µ**m. (B) Sphere-forming efficiency of rhabdospheres over three serial passages. The graph shows the amount of the primary, secondary (generated from dissociated primary spheres), and tertiary (generated from dissociated secondary spheres) spheres from 2000 cells. *p<0.05 *vs* primary spheres. (C) mRNA levels for the stem cell markers OCT3/4 and NANOG in rhabdospheres compared to native RD by Real Time PCR. *p<0.05. (D) Western blotting for OCT3/4 and NANOG in rhabdospheres compared to RD native cells (left, representative images) and densitometric analysis (right; *p<0.05). (E) Differentiation assays of rhabdospheres after incubation with appropriate differentiating stimuli. Left: osteogenic differentiation evaluated by Alizarin Red S staining, scale bar 100** µ**m; middle: adipogenic differentiation evaluated by Oil-Red-O lipid staining, scale bar 10** µ**m; right: chondrogenic differentiation evaluated by Alcian Blue staining, scale bar 50** µ**m. Representative images. (F) Transwell chemotaxis assay of rhabdospheres *vs* native RD. The graph shows the number of migrated cells in five X20 fields after 8****h. *p<0.05. (G) mRNA levels for MMP9 in rhabdospheres *vs* native RD by Real Time PCR. *p<0.05. (H) MMPs activity in the supernatant of rhabdospheres *vs* RD native cells by gelatin quenching assay. *p<0.05. (I) mRNA levels for CXCR4 in rhabdospheres compared to native RD by Real Time PCR. *p<0.05. (L) Cytofluorimetric analysis of CXCR4-positive cell fraction in rhabdospheres and native RD. Representative intensity plots for rhabdospheres and native RD (left) and percentage of CXCR4-positive cells (right). **p<0.001.

After 2**–**3 weeks of culture in growth factor-enriched serum-starved medium, RD cells formed cultures consisting in floating cell aggregates, the so-called rhabdospheres, that could be further expanded *in* **** *vitro* (Figure 1A). To assess the ability of rhabdosphere cells to initiate self-renewal, the number of spheres formed over three serial passages at each passage was determined. The number of spheres obtained at the third generation was significantly higher, indicating that rhabdospheres can be serially enriched (Figure 1B; p = 0.0495 *vs* passage 1).

### Stemness features of rhabdospheres

To assess the stem cell-related characteristics of rhabdospheres, the level of expression of two stemness markers, the transcription factors OCT3/4 and NANOG, was evaluated and compared to native RD cells. The transcription of both genes was upregulated in rhabdospheres (Figure 1C). In particular, we observed a significant increase for NANOG mRNA and a trend of increase, although not significant, for OCT3/4 (Figure 1C; p = 0.0283). Similar results were obtained for NANOG protein by Western Blot analysis, by which we detected a weak but significant increase for spheres (Figure 1D; p = 0.0495), whereas we did not find difference for OCT3/4. Notably, we also found that CD133 was not associated with the stem-like phenotype (45.9±3.4% for RMS CSC *vs* 43.8±1.8% for RD, respectively). Finally, we showed the stem cell plasticity of rhabdospheres by their ability to differentiate along three mesenchymal lineages, as demonstrated by Alizarin Red S (osteogenesis), Oil-Red-O (adipogenesis), and Alcian Blue (chondrogenesis) staining (Figure 1E).

### Enhanced migration and invasion properties of rhabdospheres

The number of rhabdosphere cells that migrated through the Boyden Chamber was significantly higher than RD (Figure 1F; p = 0.0162). Rhabdosphere cells also showed a marked *in vitro* extracellular matrix degradation potential compared to native RD, as shown by the upregulation of MMP9 mRNA (Figure 1G; p = 0.0495), and by the higher levels of gelatin degradation activity of secreted MMP9 (Figure 1H; p = 0.0368). In addition, rhabdospheres expressed very high levels of the cell surface receptor CXCR4, as demonstrated by Real Time PCR (Figure 1I; p = 0.0495) and flow cytometry (Figure 1L; p = 0.0027). This chemokine receptor mediates the migration of tumor cells towards its ligand expressing tissues [27].

### Reduced sensitivity to DXR through MDR1-independent mechanism in rhabdospheres

**Figure 2:**
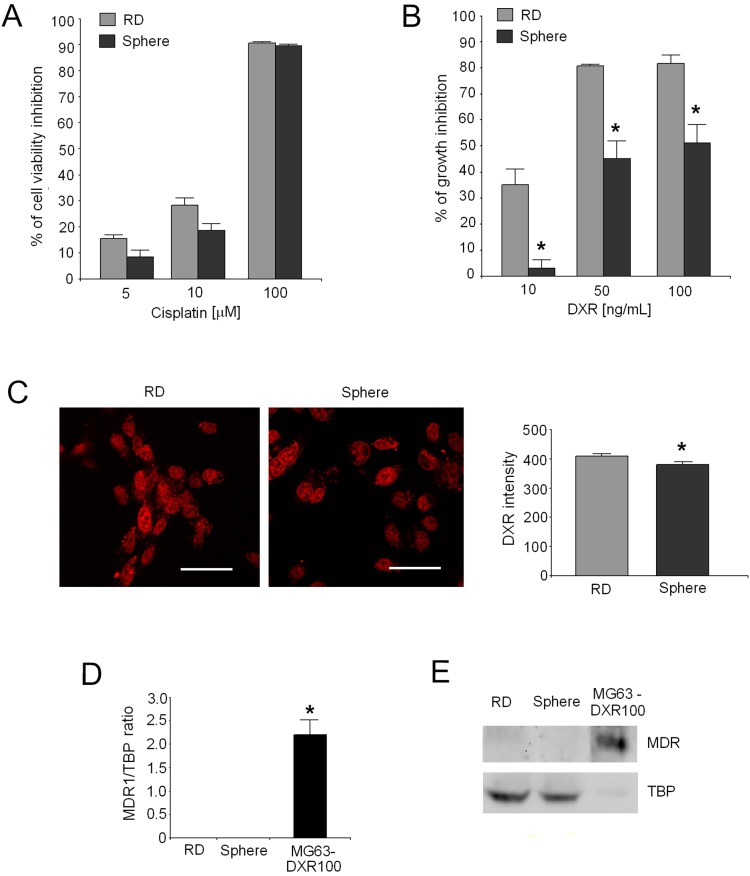
Analysis of chemoresistance of rhabdospheres. (A) Percentage of cell viability inhibition of rhabdospheres compared to native RD after treatment with different doses of cisplatin, calculated *vs* untreated cells (B) Percentage of cell growth inhibition of rhabdospheres compared to native RD after treatment with different doses of DXR, calculated *vs* untreated cells *p<0.05. (C) DXR nuclear uptake by confocal microscopy (left, representative images, scale bar 50** µ**m) and quantification of the intensity level of nuclear signal by image analysis (right; *p<0.05). (D) mRNA levels for MDR1 by Real Time PCR (*p<0.05) and (E) Western blotting for MDR1 protein expression (representative image). Rhabdospheres *vs* native RD, MG63-DXR100 multidrug resistant cells as positive control. (F) ATP intracellular content evaluated in rhabdospheres in comparison to native RD cells.

To evaluate chemoresistance in RMS CSC, rhabdospheres and native RD were exposed to increasing concentrations of cisplatin or DXR. In terms of viability inhibition, both cell populations showed the same pattern in response to cisplatin (Figure 2A, EC_50_ values: 18.7 µM for spheres and 14.6 µM for RD). On the other hand, unlike native RD, RMS CSC showed a significant lower growth inhibition in response to DXR (Figure 2B; p = 0.018 for 10 ng/mL, p = 0.0209 for 50 ng/mL, and p = 0.0202 for 100 ng/mL). EC_50_ value was 4.5-fold higher for rhabdospheres than for RD (79.5 ng/mL and 17.6 ng/mL, respectively). The intensity of the fluorescence signal of DXR in the nuclei was then evaluated to deeper investigate the chemoresistance features of rhabdospheres [28]. Although we still found nuclear localization of DXR in CSC (Figure 2C, left) that is usually associated with sensitive cells, the intensity of nuclear signal was significantly lower in rhabdospheres compared to native RD (Figure 2C, right; p = 0.0013). To investigate if this reduced nuclear concentration was due to a multidrug resistance mechanism, we analysed the expression of MDR1. We used DXR-resistant MG-63-DXR100 cells as a positive control. MDR1 mRNA (Figure 2D; p = 0.0339 MG-63-DXR100 *vs* RD and p = 0.0253 MG-63-DXR100 *vs* spheres) and P-glycoprotein (Figure 2E) were both undetectable in rhabdospheres and native RD cells, suggesting the existence of a different mechanism for the reduced sensitivity to DXR in these cells.

### Lysosomal pH in rhabdospheres

**Figure 3:**
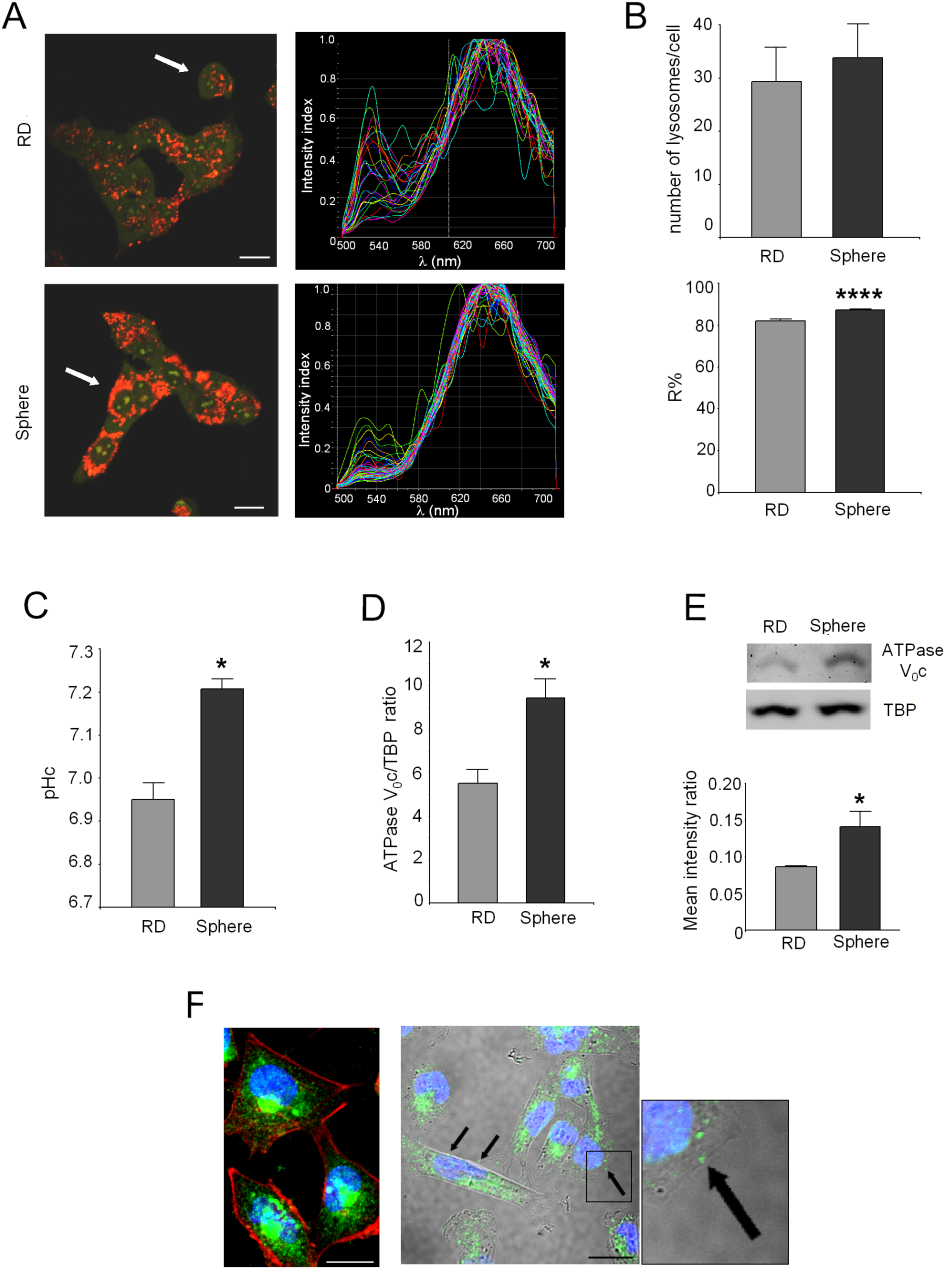
Increased lysosome acidity and V-ATPase expression in rhabdospheres. (A) AO uptake in rhabdospheres *vs* native RD by confocal microscopy. Red staining is associated with acidic vesicles, whereas green staining is associated with high pH. Representative images of AO staining (left; scale bar 50** µ**m) and emission spectra graphs (right) of lysosomes within the cells indicated in the left panel by the white arrows. X-axis, wavelength (λ); Y-axis, intensity index (max = 1). (B) Total number of lysosomes (top) and quantification of the red band contribution (R%) (bottom; ****p<0.0001) after AO staining in rhabdospheres and native RD. (C) Quantitative analysis of pHc through carboxy-SNARF-1. *p<0.05. (D) mRNA levels for ATPase V_0_c by Real Time PCR (p<0.05). (E) Western blotting for ATPase V_0_c (top, representative image) and densitometric analysis (bottom, *p<0.05). (F) Confocal analysis of rhabdosphere cells after immunofluorescence staining of ATPase V_0_a1 subunit localization (green) in the vesicular compartment (cytoskeleton marked by phalloidin-TRITC, middle) or in the cytoplasmic membrane (arrows in the bright field, left). The squared detail of plasmatic membrane localization is enlarged (right). Nuclei were counterstained with Hoechst 33258. Representative images of an xy field, scale bar 20** µ**m.

To determine lysosome pH, rhabdospheres and native RD cells were exposed to AO, a fluorescent dye that selectively accumulates into acidic vesicles in a pH-dependent manner. As shown by representative pictures (Figure 3A, left) and by the emission spectra profile of lysosomal AO (green and red emission intensities at 520–540 nm and 645–665 nm, respectively) (Figure 3A, right), AO uptake was different in RD and rhabdospheres. Quantitative analysis revealed that rhabdospheres had a higher number of acidic vesicles (Figure 3B, top) and a highly significant lower vesicular pH compared to native RD cells (Figure 3B, bottom; p<0.0001). On the contrary, a higher level of cytosolic alkalinization was detected in rhabdospheres (Figure 3C; p = 0.0495). This was possibly as a consequence of increased proton storage within the lysosomal compartment. Since V-ATPase is a key effector of vesicle acidification, we next assessed the level of the V_0_c subunit that was higher both at the mRNA (nearly 1.8-fold higher, Figure 3D; p = 0.0283) and at the protein level (Figure 3E; p = 0.0495) than in native RD. In rhabdospheres, V-ATPase was predominantly localized in the perinuclear zone, close to the nuclei, possibly within the endoplasmic reticulum (Figure 3F, left), and only little localization was observed on the plasma membrane (Figure 3F, right; arrows).

### Effect of lysosomal or V-ATPase targeting on rhabdosphere survival

**Figure 4:**
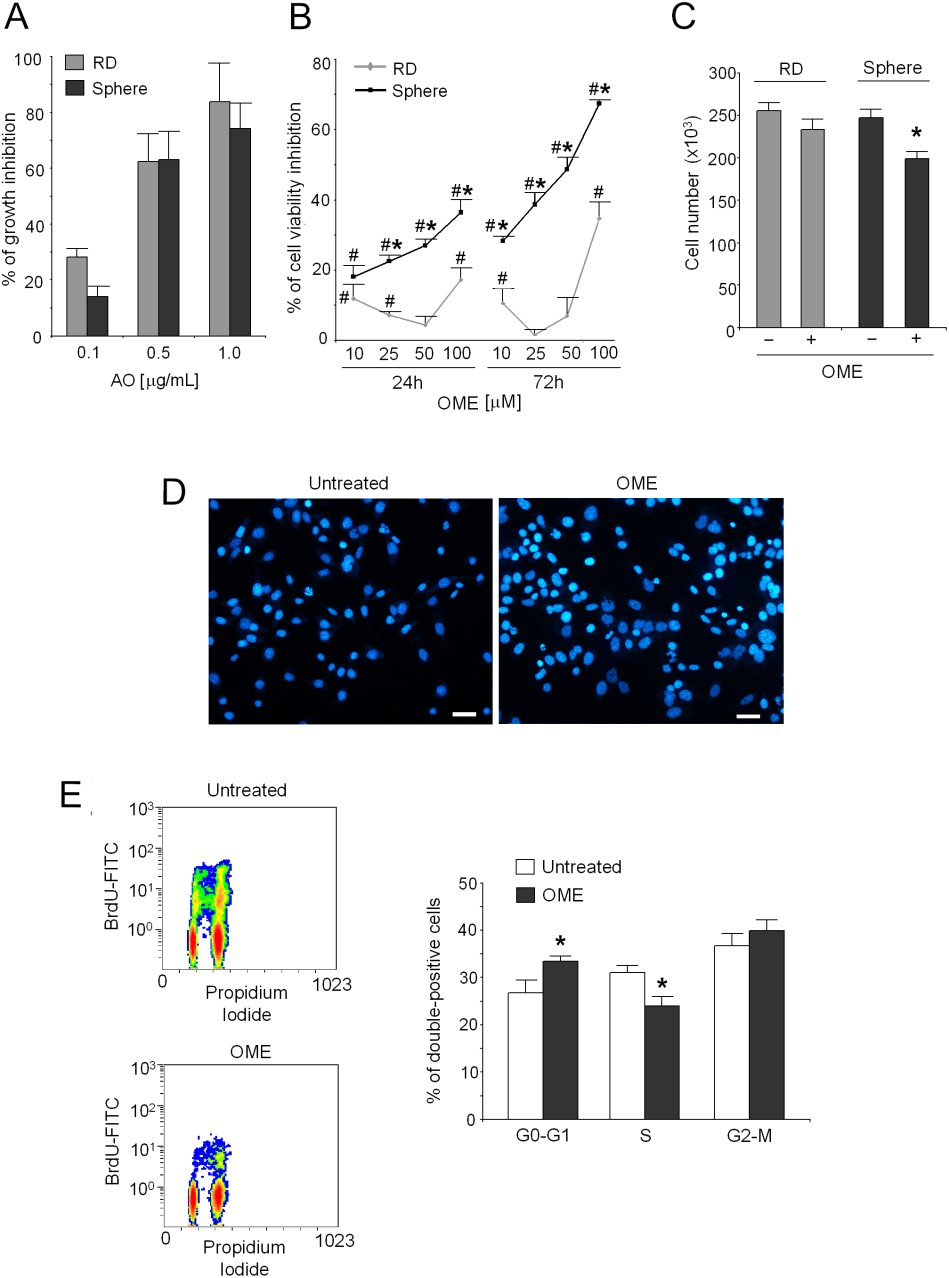
Effects of strategies targeting V-ATPase in rhabdospheres. (A) Percentage of cell growth inhibition of rhabdospheres and RD cells after treatment with AO at different concentrations evaluated by viable cell counting with respect to untreated cells. (B) Percentage of cell viability inhibition after treatment with OME at different concentrations with respect to untreated cells, evaluated by the acid phosphatase indirect assay (*p<0.05 spheres *vs* RD and #p<0.05 spheres or RD *vs* untreated). (C) Cell number evaluated by dye exclusion assay after OME treatment. *p<0.05. (D) Hoechst 33258 staining to evaluate apoptosis of rhabdosphere cells after OME treatment. Representative pictures. Scale bar 50** µ**m. (E) Cell cycle distribution of rhabdosphere cells by flow cytometry after OME treatment. Left, representative images of double stained cells indicating the total content of DNA (Propidium Iodide, X-axis) and Bromodeoxyuridine (BrdU) incorporation into newly synthesized DNA by proliferating cells during S-phase (BrdU-FITC, Y-axis). Right, graph of the percentages (*p = <0.05).

On the contrary to what obtained with DXR that was more cytotoxic for RD cells (Figure 2B), either AO (Figure 4A) or OME treatments (Figure 4B; p* = *0.0139 *vs* untreated condition) were effective in both the cell populations to inhibit the cell growth and viability, respectively. Notably, OME inhibition was even stronger in the CSC fraction. Indeed, treatment with OME affected rhabdosphere viability already at low concentrations (Figure 4B; p = 0.0202 for 25 µM at 24 h; p = 0.0209 for other conditions *vs* RD). To ascertain if the effect of OME on cell viability was a consequence of growth inhibition, or of an induction of apoptosis, we performed cell counting, cell cycle and apoptosis analyses. The number of cells was significantly decreased already after 24 h of treatment with 50 µM (Figure 4C; p = 0.0283). Adversely, after 48 h of treatment with OME 100 µM, the nuclear staining with Hoechst 33258 did not reveal evidence of apoptotic bodies (Figure 4D). On the other hand, treatment with OME 100 µM induced a significantly increased in the number of cells in G0–G1 phase, with a corresponding decrease in the S phase (Figure 4E; p = 0.0163).

### V-ATPase in CSC from other tumor histotypes

**Figure 5:**
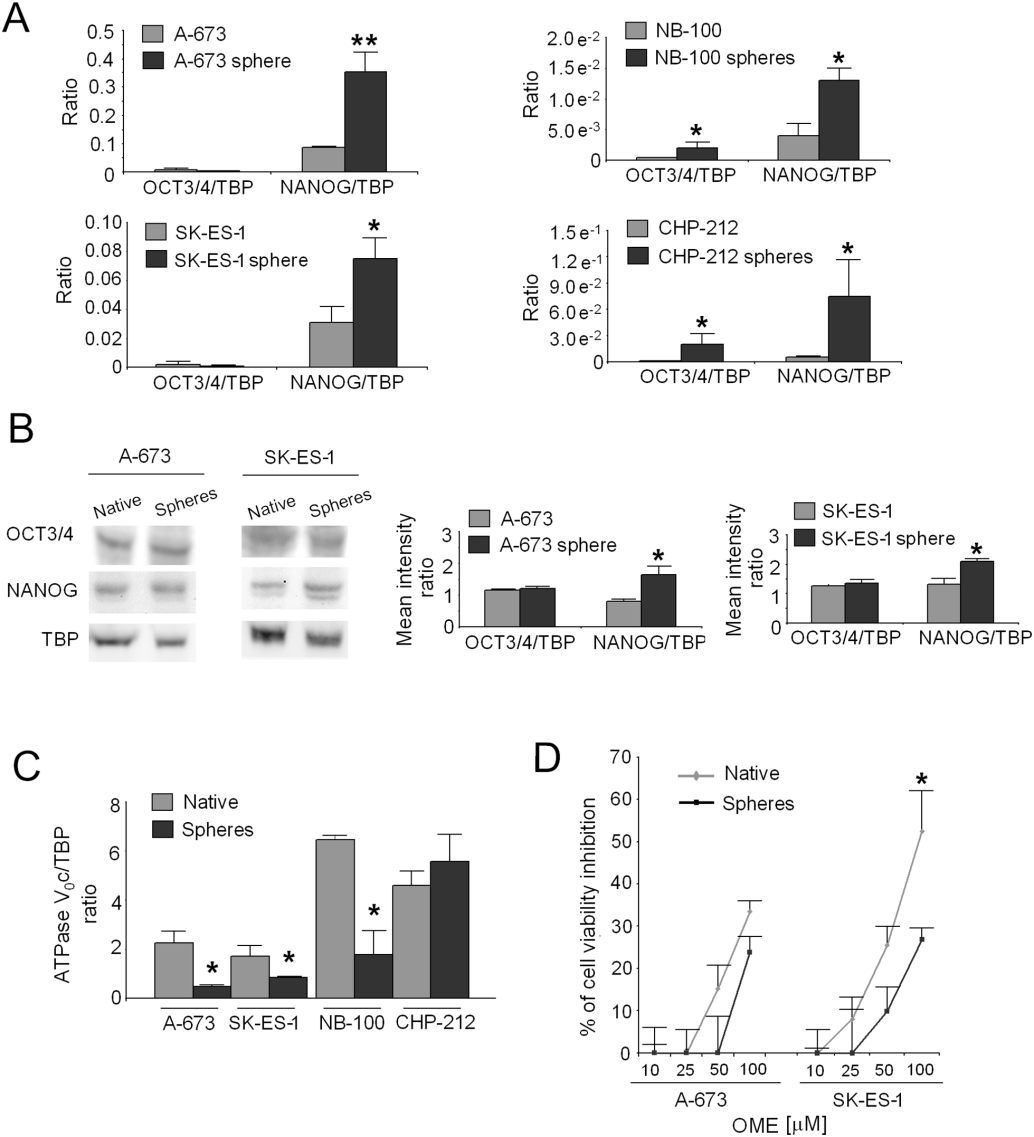
Analysis of V-ATPase expression and OME effectiveness in other CSC models. (A) Real Time PCR analysis of mRNA levels for the stem cell markers OCT3/4 and NANOG in spheres obtained from A-673, SK-ES-1 (ES, left; *p<0.05, **p<0.01), NB-100, and CHP-212 (NB, right; *p<0.05) cell lines in comparison to native cells. *p<0.05. (B) Western blotting for OCT3/4 and NANOG in ES spheres compared to native cells (left, representative images) and densitometric analysis (right; *p<0.05). (C) mRNA levels for ATPase V_0_c by Real Time PCR in ES and NB spheres compared to native cells (*p<0.05). (D) Percentage of inhibition of cell viability after 72****h of treatment of ES spheres with OME, evaluated by the acid phosphatase indirect assay with respect to untreated cells (*p<0.05).

To evaluate if the increased expression of V-ATPase is a general phenomenon that can be associated with CSC of other tumor histotypes, we successfully isolated spheres from ES (A-673 and SK-ES-1) and NB (NB-100 and CHP-212) cell lines, whereas SH-SY5Y spheres failed to growth. Although no difference was observed for the stemness marker OCT3/4, the mRNA level of NANOG was significantly increased in ES spheres compared to native cells (Figure 5A, left; p = 0,0065 for A-673 and p = 0.05 for SK-ES-1). In NB, mRNA for both OCT3/4 and NANOG was significantly higher in spheres than in native cells (Figure 5A, right; p = 0.0495). The result obtained in ES cultures was also confirmed by western blotting only for NANOG, that appeared to be weak but significantly increased in the sphere fraction (Figure 5B; p = 0.0495). Unexpectedly, in contrast to the results obtained for RMS, the mRNA level for the V_0_c ATPase was significantly reduced in spheres compared to native cells in all models but NB-100 (Figure 5C; p = 0.0209 for ES and p = 0.0495 for NB). Accordingly, the treatment with OME was not able to inhibit the growth of ES cells (Figure 5D; p = 0.0209).

### Effect of V-ATPase targeting on cancer invasiveness and sensitivity to chemotherapy drugs

**Figure 6:**
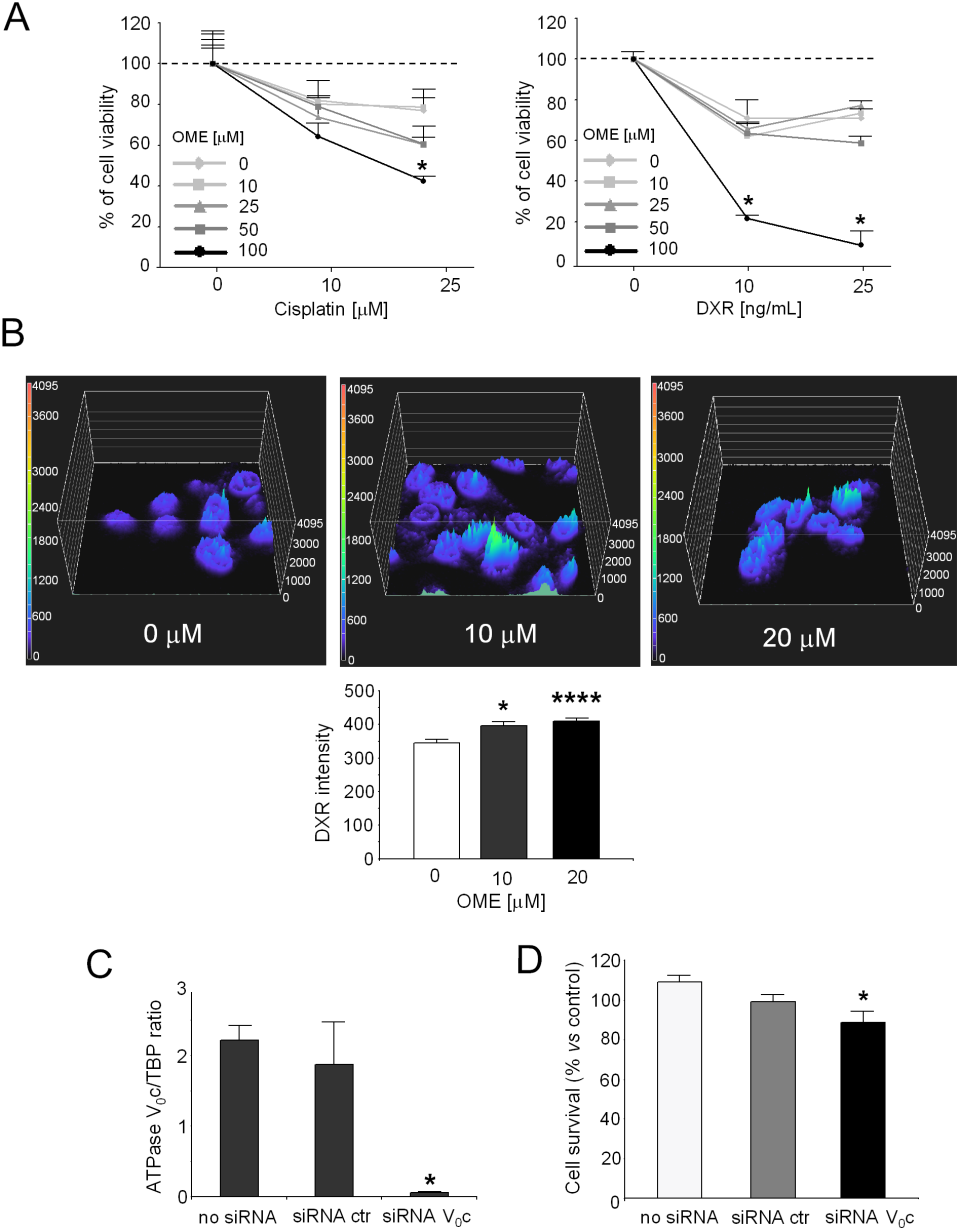
Effects of OME on chemoresistance of rhabdospheres. (A) Percentage of growth inhibition of rhabdospheres pre-treated with OME and incubated with DXR (left; *p<0.05 *vs* DXR alone-treated cells) or cisplatin (right) with respect to untreated cells, evaluated by the acid phosphatase indirect assay. (B) Confocal microscope analysis and quantification of DXR uptake in rhabdospheres after OME pre-treatment. Nuclear DXR fluorescence intensity indicated with colour and height in surface intensity plots (representative images, top), and quantification of nuclear signal by bar graph (bottom; *p<0.05 for 10** µ**M and ****p<0.0001 for 20** µ**M). (C) mRNA analysis for ATPase V_0_c after rhabdosphere electroporation with a specific siRNA against the V_0_c subunit. (D) Cell survival of rhabdospheres electroporated with the specific siRNA against ATPase V_0_c and treated with 25****ng/mL of DXR, determined by the acid phosphatase indirect assay (*p<0.05 *vs* no siRNA).

Rhabdospheres were pre-treated with 10 to 100 µM of OME for 24 h, and then exposed to low doses of DXR or of cisplatin for additional 48 h. We observed that the addition of OME to cisplatin treatment was only mildly effective when compared to cells treated with cisplatin alone (Figure 6A, left). On the contrary, only pre-treatment with 100 µM of OME significantly enhanced the toxicity of both the doses tested of DXR (Figure 6A, right; p = 0.0209). Moreover, after 1 h of pre-treatment with different concentrations of OME followed by direct observation at confocal microscope, we found a significant increase of nuclear localization of DXR, as revealed by the presence of bright peaks in the surface intensity plots (Figure 6B, top), and by image analysis quantification (Figure 6B, bottom; p = 0.016 for 10 µM and p<0.0001 for 20 µM). This result suggests that inhibition of V-ATPase induces an increase of intracellular DXR, in turn, leading to an increase of its nuclear concentration and cytotoxicity. The involvement of V-ATPase in the sensitivity of rhabdosphere to DXR was confirmed by the use of siRNA transcriptional gene silencing of V_0_c (Figure 6C; p = 0.0495 *vs* no siRNA) that, like OME, significantly increased DXR cytotoxicity (Figure 6D; p = 0.0209 *vs* no siRNA). The extent of the inhibition was lower in respect to the treatment with OME because, for this assay, we could not use acidic conditions. In fact, electroporation increases the cell membrane permeability to the extracellular excess of protons, ultimately leading to a strong unspecific cytotoxic effect. Nevertheless, the role of V-ATPase in CSC is mainly important under acidic conditions.

**Figure 7:**
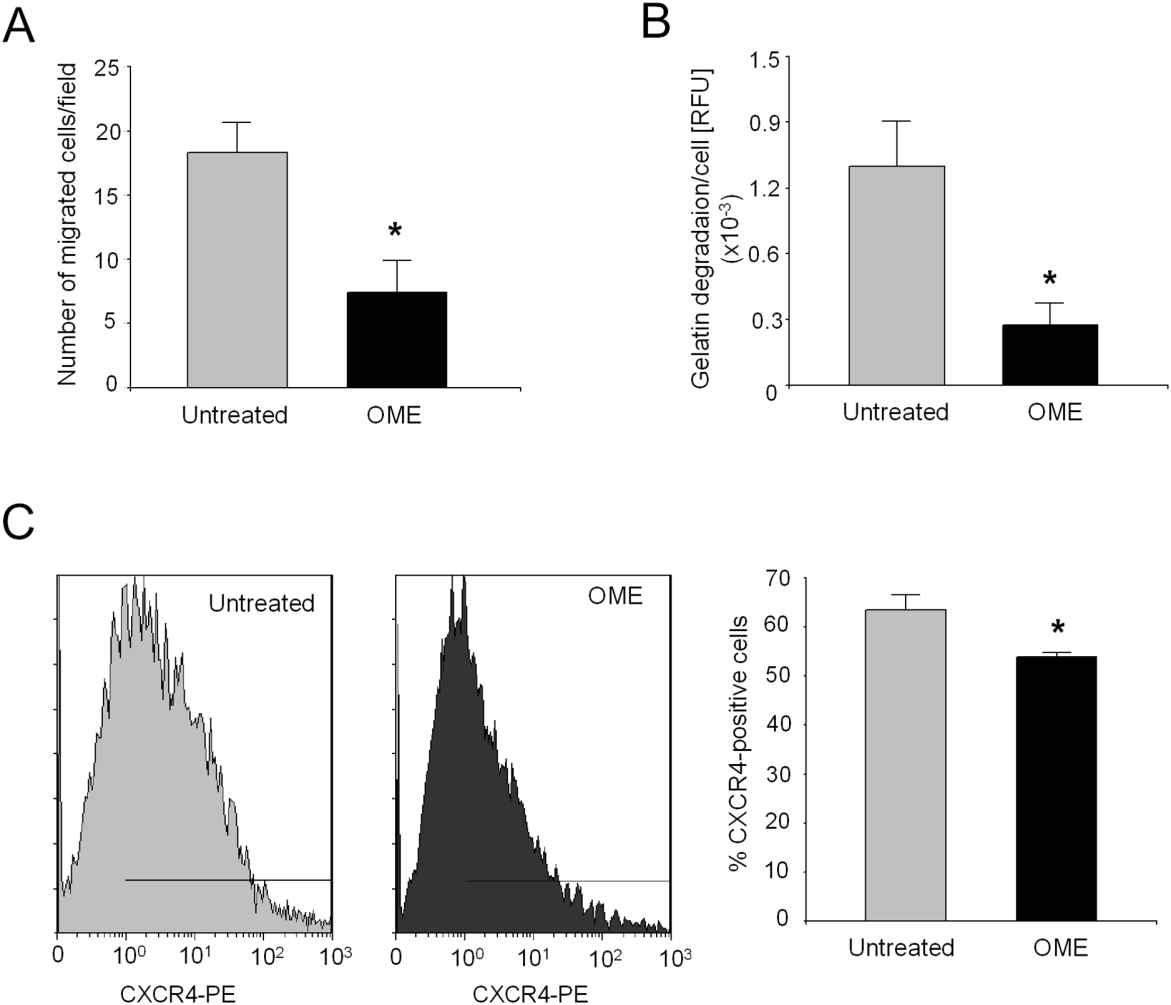
Effects of OME on invasiveness of rhabdospheres. (A) Transwell chemotaxis assay of rhabdospheres after pre-treatment with OME. *p<0.05. (B) MMPs activity in the supernatant of rhabdospheres after pre-treatment with OME by gelatin quenching assay. *p<0.05. (C) Cytofluorimetric analysis of CXCR4-positive cell fraction in rhabdospheres after treatment with OME. Left, representative intensity plots. Right, graph of the percentages. *p<0.05.

The *in vitro* migration and invasive potential of rhabdospheres after exposure to OME was also investigated. The treatment of 24 h with OME significantly reduced the number of migrated cells (Figure 7A; p = 0.0247), and the invasive potential of rhabdospheres, evaluated in terms of MMPs secretion (Figure 7B; p = 0.0494). Furthermore, the treatment with 100 µM of OME significantly decreased the expression of the chemokine receptor CXCR4, closely associated with tumor migratory ability (Figure 7C; p = 0.0209).

## Discussion

Approximately one-third of RMS patients without metastases at presentation and treated with chemotherapy, in combination with surgery and/or radiation therapy, undergo clinical progression [2], suggesting the presence of a reservoir of sarcoma cells that do not respond to anticancer agents. This reservoir might reside in the quiescent or slowly-proliferating cell subset, known as CSC, that features self-renewal potency and the ability to give rise to highly proliferating elements, and that are widely recognised as a key player in clinical drug resistance and tumor relapse [3]. The presence of chemoresistant CSC has been already described in RMS [14], [15]. In order to improve the outcome of high-risk RMS patients, the mechanisms responsible for a reduced effectiveness of anticancer agents in RMS CSC are currently a subject of great investigation.

To obtain RMS CSC-enriched cultures, we used the sphere system method that is based on the ability of CSC to grow as spherical colonies under low-adherence conditions (rhabdospheres), a widely accepted feature of CSC in solid tumors [6] [29]. As a proof of the enrichment in CSC-like cells of rhabdosphere cultures, we demonstrated in the higher levels of expression of the stemness-related factors OCT3/4 and NANOG in rhabdospheres as compared to native cells. Rhabdospheres could also be serially enriched and differentiated into multiple mesenchymal lineages, as further index of self-renewal ability and plasticity typical of stem-like cells. Then, to deeply characterize RMS CSC, we tested their *in vitro* invasiveness and migration potential. We performed a chemotaxis assay and we looked to MMP9 and CXCR4 expression. MMP9 is a protease is involved in the breakdown of extracellular matrix and that has been associated with CSC in solid tumors [30]. CXCR4 is a chemokine receptor for the stromal-derived factor 1 that plays an important role in the induction of chemotactic and invasive responses in several solid tumors [27], including osteosarcoma [31] and RMS [32], [33]. Rhabdospheres showed higher MMP9 expression and activity, higher migration ability, and increased CXCR4-positive cell fraction in comparison to native cells. Our results confirmed a striking attitude of CSC to invade the surrounding environment.

A pivotal feature of CSC is the resistance to anticancer drugs [9]. Indeed, the ability to extrude nuclear dyes such as Hoechst 33258 via ABC transporters has been extensively used as a tool to characterize CSC [14], [34]. For this reason, we tested the cytotoxic activity of two drugs currently used in RMS treatment, cisplatin and DXR. Cisplatin was equally effective in RMS CSC and native cells. Regarding DXR, although we did not observe a chemoresistant phenotype that is usually associated with the lack of nuclear localization of DXR, RMS CSC showed a lower amount of DXR accumulation as well as a significantly lower *in vitro* drug sensitivity compared to native cells. The reduced sensitivity to DXR was not due to an increased expression of P-glycoprotein, as it might be expected based on the well-established role of multidrug resistance in human sarcomas [35], [36]. We therefore hypothesised that the lower sensitivity observed in RMS CSC compared to native cells was a consequence of an aberrant vesicle acidification status. In fact, a high acidity of lysosomes can increase the trapping of weakly basic drugs, like DXR drug, inside the lysosomal compartment, the so called “ion trapping” mechanism, and allows cancer cells to develop chemoresistance [37], [38]. As a confirmation, lysosomal pH was found to be significantly lower in RMS CSC, whereas cytosolic pH was higher as compared to native cells. An aberrant ion pumping mediated by proton transporters may be responsible not only for an increased lysosomal acidity, but also for an altered pH gradient across the plasma membrane that can, in turn, obstacle the DXR intracellular uptake also by an alternative mechanism, the alteration of the surface pressure and permeability of the cytoplasmic membrane, like elegantly demonstrated by other authors [39]. Both the suggested mechanisms would indirectly and ultimately lead to a decreased concentration of DXR in the nucleus, a phenomenon that we demonstrated in our model. More acidic lysosomes can be also responsible for an increase of cancer invasiveness through the release and activation of MMPs during the invasion process. Indeed, an increase of lysosomal diameter, together with a decrease of luminal pH, has already been observed in highly metastatic breast cancer cell lines [18], and in chemoresistant neoplastic cells [40]. This study is the first experimental evidence of the role of vesicular acidification in CSC biology.

To preliminarily investigate if the targeting of lysosomal activity could be a valuable approach to affect CSC, we treated RMS CSC with AO. This is a nontoxic cationic dye that strongly accumulates in acidic compartments, such as lysosomes. AO has been introduced as a tool for the photodynamic treatment of sarcomas due to its selective accumulation in tumor environment, and its antineoplastic activity has been extensively validated in sarcoma cells [25], [41]. We found that, in contrast to DXR, AO treatment successfully targets RMS CSC. These findings demonstrated that lysosomal targeting is a successful strategy to affect CSC in RMS.

To more completely describe this phenomenon in rhabdospheres, we then explored the role of V-ATPase that is the main protein responsible for the regulation of lysosomal pH [42]. V-ATPases are large, multi-subunit complexes organized into two domains, the peripheral domain (V_1_) that carries out ATP hydrolysis, and an integral domain (V_0_) responsible for exchanging protons. A high expression of this pump has been associated with chemoresistance and metastatic behaviour, and V-ATPase targeting is being introduced as a promising therapeutic tool [18], [43]. We focused on the V_0_c subunit that has been associated with chemoresistance [44] and that, specifically in RMS, modulates cell survival [24]. Looking at the expression of this subunit, we found that both mRNA and protein are significantly upregulated in RMS CSC compared to native cells, and that the protein is particularly expressed at the perinuclear region, where it is able to protect the nucleus from an excess amount of protons [17]. As a following step, we looked at more specific therapies targeting the V-ATPase in the CSC population. Thus, we explored the use of OME, a PPI that selectively inhibits V-ATPase through the binding to the V_1_a subunit [26] and, as we also previously demonstrated, is able to alkalinize acidic lysosomes and to impair sarcoma cell survival [22], [24]. Interestingly, administration of OME was even more effective in RMS CSC than in native cells. To date, the mechanism of toxicity of not photoactivated AO is not clear, and therefore we do not know why OME was more effective than AO in RMS CSC. However, we deeper investigated on the mechanism on the basis of the inhibitory mechanisms of OME, and we observed a reduction of S phase and an increase in G0–G1 in CSC, without evidence of apoptosis, suggesting a cytostatic rather than cytotoxic effect for this drug. The involvement of lysosomes in cell growth was not unexpected. Indeed, these organelles have already been associated to the loss of control of the cell growth, to a deregulation of cell death, and to the acquisition of chemoresistance and metastatic potential [45]. Lysosomes are the most important storage compartment for proteases and other hydrolytic enzymes, and V-ATPase is primarily involved in the maintenance of cellular homeostasis by accomplishing the degradation of autologous material. This is of particular importance for metastatic CSC since, once outstripped of the blood supply, run into an unfavourable hypoxic context becoming oxygen-and nutrient-starved [46]. Therefore, starved CSC are forced to activate catabolic processes useful for the recycling of intracellular components, as an alternative source of energy to maintain homeostasis, quiescence and viability during metabolic stress.

To verify if such promising therapeutic approach can be also proposed for other paediatric solid cancers, we analysed the level of expression of V-ATPase in CSC obtained from Ewing’s sarcoma and neuroblastoma. However, in these CSC cultures, we did not find higher levels of V-ATPase than in native cells and, consequently, we did not observe an increased sensitivity to OME, concluding that the mechanism here proposed and mediated by V-ATPase is specific for the CSC of RMS histotype.

On the light of the concept that lysosomes are involved in RMS drug resistance, we then evaluated if the treatment with OME could enhance DXR cytotoxicity in RMS CSC. We found that pre-treatment with OME did not improve the effect of cisplatin, but induced an increase in the nuclear accumulation of DXR, in turn resulting in a significantly higher cytotoxic effect. This additional result confirms that the less sensitive phenotype to DXR in RMS CSC is dependent on V-ATPase function.

To further directly correlate the V-ATPase activity with a low DXR response, we impaired the expression of the proton pump by a specific silencing approach. Indeed, this strategy resulted in a strong decrease of V-ATPase mRNA expression and was able to partially restore the sensitivity of RMS CSC to DXR.

Interestingly, beyond the ability to restore CSC sensitivity to DXR, OME treatment was also able to impair the high *in vitro* invasiveness of RMS CSC in terms of migration ability, MMPs release, and enrichment of a CXCR4-positive subpopulation. As mentioned above, lysosome function has been associated to metastatic spread of breast cancer [18], and a recent hypothesis has correlated lysosomes to the regulation of the migratory/invasive phenotype of breast and glioblastoma stem cells as an essential part of the autophagic flux [47].

**Figure 8:**
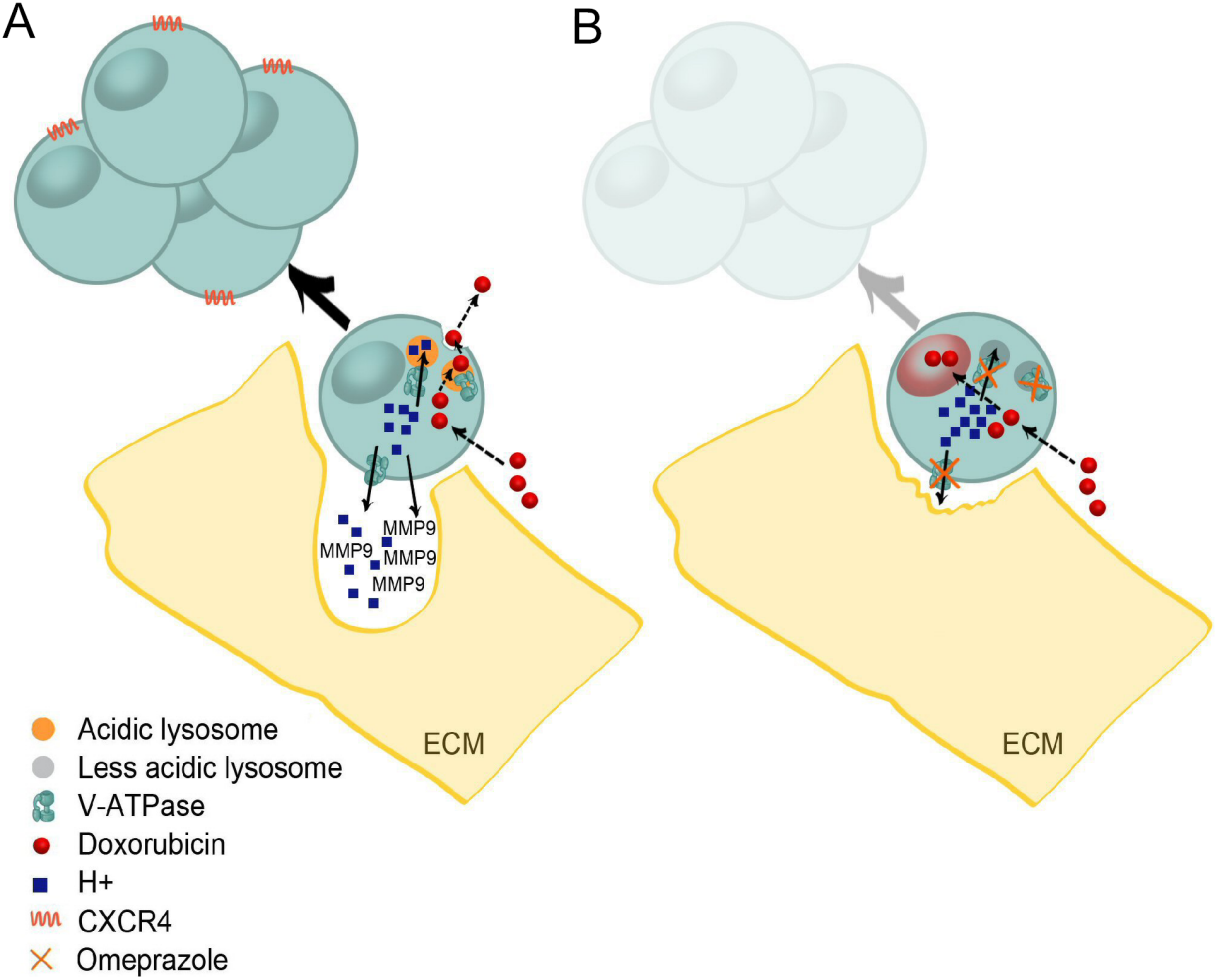
Targeting lysosomal acidity in rhabdomyosarcoma CSC. Lysosome acidification in RMS CSC is mediated by V-ATPase, and plays an important role for growth, chemoresistance, and invasiveness of these cells (A). The anti-V-ATPase OME is an effective drug and it can be proposed as a valuable strategy to affect RMS CSC (B).

Taken together, these data highlight the role of lysosomal pH in several aspects of RMS CSC biology and provide consistent evidence that vesicle acidification sustained by V-ATPase can be considered as a hallmark of RMS CSC, in which it drives mechanisms of a reduced sensitivity to anticancer drugs and activities related to invasion and metastasis (Figure 8A). Our results encourage the exploitation of new and more specific anti-cancer drugs with an increased toxicity to CSC and reduced unfavourable side effects through the targeting of the lysosomal activity (Figure 8B). Photodynamic therapies that take advantage of low pH of tumor cells [48], the use of PPI as anticancer drugs [49], and the design of novel nanocarriers based on pH sensitive lipids or polymers [50] demonstrate that this is a powerful strategy to be recommended for translation to the CSC subpopulation.
